# A novel feature fusion network for multimodal emotion recognition from EEG and eye movement signals

**DOI:** 10.3389/fnins.2023.1234162

**Published:** 2023-08-03

**Authors:** Baole Fu, Chunrui Gu, Ming Fu, Yuxiao Xia, Yinhua Liu

**Affiliations:** ^1^School of Automation, Qingdao University, Qingdao, China; ^2^Institute for Future, Qingdao University, Qingdao, China; ^3^Shandong Key Laboratory of Industrial Control Technology, Qingdao, China

**Keywords:** multimodal emotion recognition, electroencephalogram (EEG), eye movement, feature fusion, multi-scale, Convolutional Neural Networks (CNN)

## Abstract

Emotion recognition is a challenging task, and the use of multimodal fusion methods for emotion recognition has become a trend. Fusion vectors can provide a more comprehensive representation of changes in the subject's emotional state, leading to more accurate emotion recognition results. Different fusion inputs or feature fusion methods have varying effects on the final fusion outcome. In this paper, we propose a novel Multimodal Feature Fusion Neural Network model (MFFNN) that effectively extracts complementary information from eye movement signals and performs feature fusion with EEG signals. We construct a dual-branch feature extraction module to extract features from both modalities while ensuring temporal alignment. A multi-scale feature fusion module is introduced, which utilizes cross-channel soft attention to adaptively select information from different spatial scales, enabling the acquisition of features at different spatial scales for effective fusion. We conduct experiments on the publicly available SEED-IV dataset, and our model achieves an accuracy of 87.32% in recognizing four emotions (happiness, sadness, fear, and neutrality). The results demonstrate that the proposed model can better explore complementary information from EEG and eye movement signals, thereby improving accuracy, and stability in emotion recognition.

## 1. Introduction

Emotions are influenced by various factors, and different emotions manifest themselves through facial expressions and tone of voice, among other aspects. Emotion is an integral part of intelligence and cannot be separated from it. Therefore, the next breakthrough in the field of artificial intelligence may involve endowing computers with the ability to perceive, understand, and regulate emotions. Professor Picard and her team at the Massachusetts Institute of Technology (MIT) formally introduced the concept of affective computing (Picard, 2000) and emphasized the crucial role of affective computing in human-computer interaction (Picard et al., 2001). Human emotion recognition is essential for applications such as affective computing, affective brain-computer interfaces, emotion regulation, and diagnosis of emotion-related disorders (Pan et al., 2018). Therefore, it is necessary to establish accurate models for emotion recognition.

In recent years, emotion recognition systems have primarily utilized speech signals (El Ayadi et al., 2011), facial expressions (Ko, 2018), non-physiological signals (Yadollahi et al., 2017), and physiological signals (Shu et al., 2018) for emotion recognition. Each of these modalities has its own prominent characteristics. Subjective behavioral signals (including facial expressions, speech, eye movements, etc.) are convenient to acquire, but they are influenced by various factors, such as the potential for facial expression masking. Objective physiological signals (including electroencephalogram (EEG), electrocardiogram, etc.) are less susceptible to masking and can more accurately reflect changes in a person's emotions, but their acquisition methods are more complex (Zhou et al., 2022). EEG signals have shown remarkable performance in emotion recognition (Zheng and Lu, 2015; Yang et al., 2017; Yin et al., 2017; Zheng et al., 2017), making them a suitable method for extracting human affective information.

Generally, in most cases, various features are extracted from EEG signals, and these extracted features are then utilized for classification purposes. Petrantonakis and Hadjileontiadis (2009) proposed an EEG-based feature extraction technique using higher-order crossing (HOC) analysis and implemented a robust classification approach. They tested four different classifiers and achieved efficient emotion recognition. Shi et al. (2013) introduced differential entropy (DE) features in five frequency bands for the first time and demonstrated the effectiveness of DE features in representing EEG signals. Duan et al. (2013) extracted frequency-domain features from multi-channel EEG signals in different frequency bands and employed SVM and KNN as emotion classifiers for classification purposes. However, extensive evidence suggests that traditional machine learning approaches fail to establish a direct connection between extracted features and emotional changes (Liu et al., 2019; Huang et al., 2021). To capture deeper emotional features, we employ deep learning for feature extraction in this study.

Deep learning has shown superiority over traditional machine learning methods in various fields such as computer vision (Jaderberg et al., 2015), natural language processing (Hu, 2019), and biomedical signal processing (Craik et al., 2019). Furthermore, deep learning approaches have been widely employed in emotion recognition based on EEG signals. Maheshwari et al. (2021) proposed a multi-channel deep convolutional neural network (CNN) for emotion classification using multi-channel EEG signals. Chen et al. (2019) introduced a deep CNN-based method for EEG emotion feature learning and classification, exploring the impact of temporal features, frequency-domain features, and their combinations on emotion recognition using several classifiers. Zhang et al. (2018) introduced a spatio-temporal recursive neural network (STRNN) for emotion recognition, which extracts spatio-temporal features from EEG signals for emotion recognition and achieves promising performance. Li et al. (2020) proposed a novel framework called the Bilateral Hemisphere Difference Model (BiHDM) to capture the differential information between the left and right hemispheres in EEG signals. They employed four directed Recurrent Neural Networks (RNNs) to capture the spatial information of EEG electrode signals, and a domain discriminator was utilized to generate domain-invariant emotion features. Zhang et al. (2019) proposed a design called Graph Convolution Broad Network (GCB-net), which utilizes graph convolution layers to extract features from graph-structured inputs and employs stacked regular convolution layers to capture relatively abstract features. To enhance the performance of GCB-net, a Broad Learning System (BLS) is applied to augment its capabilities. Shen et al. (2020) introduced a CRNN model that combines Convolutional Neural Networks (CNNs) with Recurrent Neural Networks and Long Short-Term Memory (LSTM) cells for extracting frequency, spatial, and temporal information from multi-channel EEG signals for emotion classification, and demonstrated the effectiveness of the model. Li et al. (2023) proposed a multi-scale Convolutional Neural Network (STC-CNN) that extracted and fused the spatio-temporal domain features and connectivity features of EEG signals for emotion classification. In the methods for extracting emotional information, Convolutional Neural Networks (CNNs) have shown promising performance (Moon et al., 2018; Khare and Bajaj, 2020). Therefore, we employ CNNs to extract emotional features from the EEG and eye movement modalities.

However, Human emotions are rich in expression, and it is not possible to accurately describe emotions using single modal signals alone. In recent years, researchers have proposed the use of multimodal signal fusion methods for emotion recognition. Schirrmeister et al. (2017) inspired by the success of deep learning in emotion recognition, proposed an emotion recognition system that combines visual and auditory modalities. This system utilizes convolutional neural networks (CNNs) to extract emotional information from speech, deep residual networks to extract visual information, and employs long short-term memory (LSTM) networks for end-to-end training. Lu et al. (2015) demonstrated the effectiveness of eye movement signals in distinguishing between different emotion categories. They also discovered that fusing EEG and eye movement signals enhances the accuracy of emotion classification, indicating a relationship between the two modalities. Zhou et al. (2022) proposed a framework for integrating subjective and objective features, which combines the spatiotemporal features of EEG signals and the gaze features. This framework aims to achieve improved emotion recognition based on EEG and eye movement signals. Mao et al. (2023) introduced a cross-modal guidance and fusion network that effectively utilizes both EEG and eye movement signals and combines them to achieve enhanced RSVP decoding performance. Fei et al. (2022) proposed a cross-modal deep learning method based on Canonical Correlation Analysis (CCA), referred to as Cross-Modal Deep CCA (CDCCA). By applying specific CCA constraints, each modality is transformed and aligned in a hyper-space. During the testing phase, only eye movement signals are used as input, while knowledge of the electroencephalogram (EEG) signals is learned during the training phase. Existing feature fusion methods mostly focus on feature selection without reflecting the distinctiveness of the features, which may result in the model not fully leveraging the strengths and weaknesses of different features.

**Figure 1:**
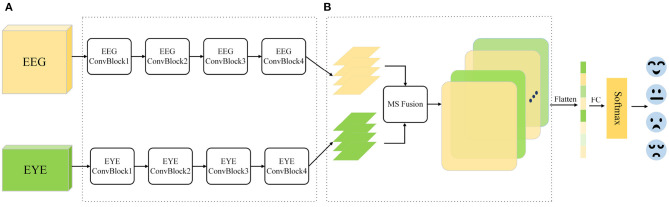
Multimodal emotion recognition framework. **(A)** Dual branch feature extraction module. **(B)** Multi-scale feature fusion module.

To address the aforementioned issues, this paper proposes a novel multimodal feature fusion neural network(MFFNN). Since the eye movement signals in the SEED-IV dataset are collected every 4 seconds, considering the rationale of fusing two modalities, we process the EEG signals using a 4-second time window. Figure 1 illustrates the multimodal emotion recognition framework proposed in this study. The main contributions of this study are as follows.

- A novel multimodal feature fusion neural network model is proposed. The dual-branch feature extraction module extracts emotional features, while the multi-scale feature fusion module explores complementary information in eye movement signals and EEG signals, selecting the most relevant features for emotion fusion.
- Experimental validation is conducted on the SEED-IV dataset, demonstrating the effectiveness of the proposed model, and validating the functionality of each module.

The remaining sections of the paper are as follows: In Section 2, a detailed description of the multimodal fusion framework is provided, including the dual-branch feature extraction module (EEG signal feature extraction, eye movement signal feature extraction) and the multi-scale feature fusion module (fusion of features from both modalities). In Section 3, the SEED-IV dataset is introduced, and experiments are conducted to analyze and discuss the experimental results, validating the feasibility and effectiveness of the model. Finally, Section 4 concludes the article.

## 2. Methodology

### 2.1. Multimodal feature fusion neural network model

Multimodal fusion enables the utilization of complementary information from different modalities to discover dependencies across modalities. The role played by each feature map extracted in the classification task may vary, necessitating further selection. In other words, certain portions of the features contain necessary information related to discriminating between target and non-target class samples, while other parts have minimal impact on the classification. Therefore, to extract the most relevant and complementary features from different modalities for emotion recognition, we propose a Multimodal Feature Fusion Neural Network (MFFNN) that selects and fuses the features from different modalities that are most correlated with emotions. The allocation of weights is determined based on the interactions within and between modalities.

The proposed MFFNN framework, as illustrated in Figure 1, consists of two main modules: the dual-branch feature extraction module and the multi-scale feature fusion module. The dual-branch feature extraction module comprises two parallel backbones responsible for extracting emotional features from both EEG and Eye modalities. A multi-layer convolutional structure is employed as the feature extractor, enabling effective extraction of emotional information from both modalities. To address the limitations of each modality and leverage their complementarity, a multi-scale feature fusion module is proposed. This module adaptively selects information from different spatial scales and employs soft attention mechanisms to explore interactions between modalities, aiming to identify the most relevant features for emotion recognition. The selected features are weighted and fused to obtain the fusion feature *F*, which is then input to a fully connected layer and activated by Softmax to yield the classification result.

**Figure 2:**
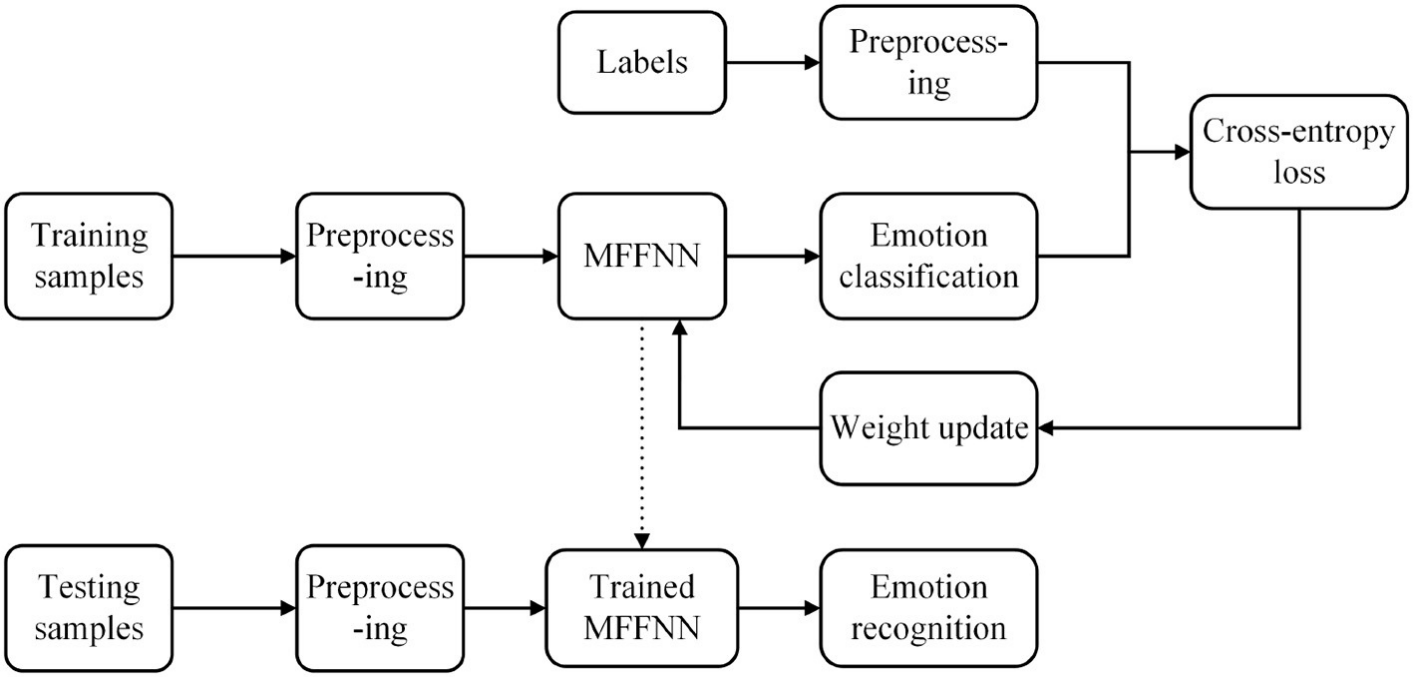
The overview of MFFNN.

As shown in Figure 2, the dataset samples are first divided into training and testing samples. Subsequently, the training and testing samples are preprocessed by removing the baseline signal. Additionally, the slice window technique is employed for label preprocessing. Next, the training samples are used to train the proposed MFFNN model, computing the cross-entropy loss, and updating the network parameters using the Adam optimizer (Kingma and Ba, 2014). Finally, the trained model is employed to recognize the emotional states of the testing samples, and the classification accuracy is used as the final recognition result.

### 2.2. Dual-branch feature extraction module

#### 2.2.1. EEG feature extraction

**Figure 3:**
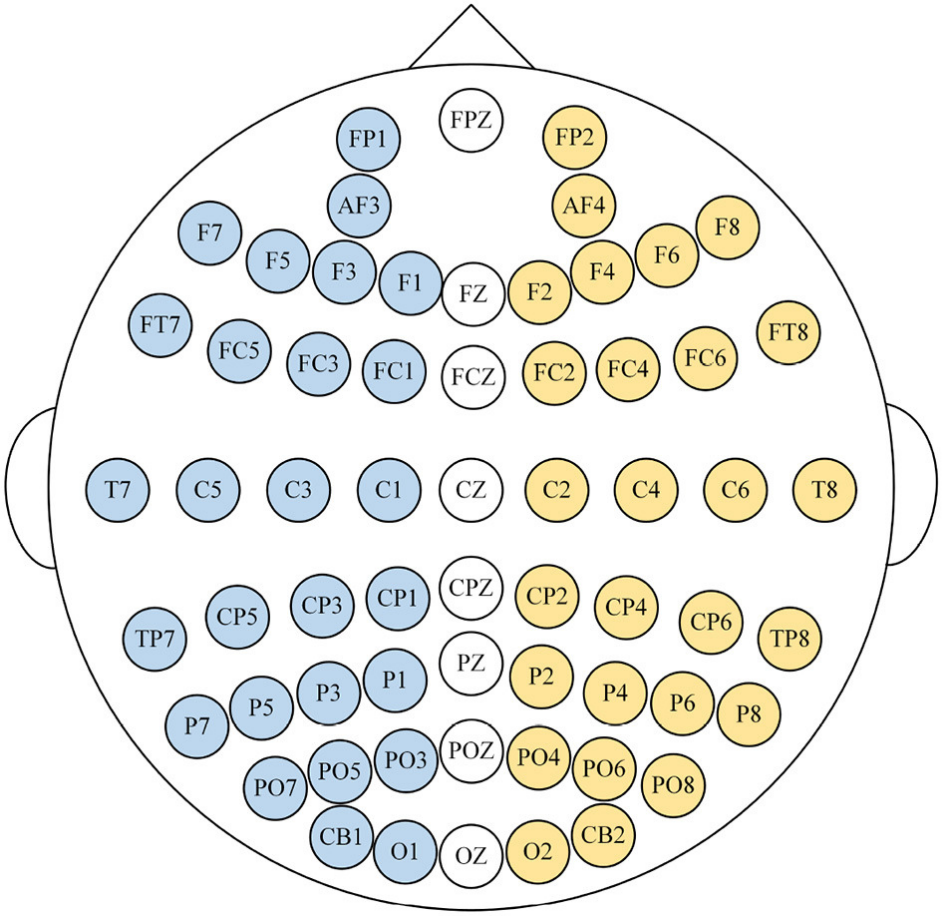
EEG electrode distribution.

**Figure 4:**
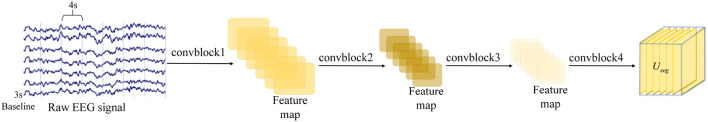
EEG spatio-temporal feature extraction structure.

To facilitate the extraction of emotional information from EEG signals, EEG signals are treated as two-dimensional data, where one dimension represents EEG channels and the other dimension represents time. The SEED-IV dataset experiment's electrode distribution is illustrated in Figure 3, with each channel corresponding to a specific brain location, providing spatial information of the EEG signal. Additionally, as emotions change over time, the EEG signals also carry temporal information. To extract features from both the temporal and spatial aspects of the EEG, we have designed our EEG feature extraction architecture based on CNN, as depicted in Figure 4.

Preprocessing of EEG data: The SEED-IV dataset provides 62-channel EEG data. In order to remove noise and eliminate artifacts, bandpass filters ranging from 1 Hz to 75 Hz were utilized to preprocess the EEG data, along with baseline correction. Considering that the eye movement signals in the SEED-IV dataset are collected every 4 seconds, and taking into account the rationality of fusing the two modalities, we divide the EEG signals into non-overlapping time windows of 4 seconds. To reduce computational complexity, we downsample the temporal dimension of the input samples to 60, resulting in EEG modality samples of shape 240 (4s × 60) ×62 (62 electrodes).

After preprocessing, to better capture the spatiotemporal characteristics of EEG signals related to emotions, our EEG feature extraction model includes four convolutional blocks. The first three convolutional blocks include a normalization layer and a max-pooling layer, while the last convolutional block does not include a max-pooling layer. Each convolutional block consists of a convolutional layer and two different convolutional kernels for extracting the spatiotemporal features of the EEG. Each individual convolutional layer employs ReLU as the activation function. The two convolutional kernels have different sizes, namely 3 × 1 and 1 × 5. The 3 × 1 kernel convolves the data along the temporal dimension to extract the temporal features of the EEG signals, while the 1 × 5 kernel convolves the data along the spatial dimension to extract the spatial features of the EEG signals.

#### 2.2.2. Eye movement feature extraction

We utilize the SEED-IV dataset as the experimental dataset in our study. This dataset encompasses detailed parameters of various eye movement signals, including pupil diameter, gaze details, saccade details, blink details, and event details statistics. Studies in neuroscience and psychobiology have shown a connection between emotions and eye movement data, particularly pupil diameter and dilation response. Therefore, we focused on investigating the changes in pupil diameter during emotional variations.

Preprocessing of EYE data: Due to the significant influence of illumination on pupil diameter, we employed a principal component analysis (PCA) method (Soleymani et al., 2011) to remove the interference of illumination on pupil diameter. The specific implementation process is as follows.

Let *M* be the matrix of *X* × *Y*_*i*_, which contains the response of the subject to the same video, *X* represents the sample, *Y*_*i*_ represents the participant, *M* is divided into two components, as shown by the following equation:


(1) $$\begin{array}{l} M=A+B \end{array}$$

*A* is the strongest illuminance response in the signal, which is the most crucial aspect. Studies have shown that the size of the pupillary light reflex varies with age, and most participants in our experiment are young, in their twenties, thus eliminating the influence of aging. *B* represents the emotional information generated after receiving video stimuli. The sources of these two components are independent, and the decorrelation of principal component analysis can separate these two components. We utilize principal component analysis (PCA) to decompose *M* into components of *Y*_*i*_. In order to capture the emotional information contained in the pupil diameter, we assume that the first principal component approximates the estimation of light reflection. Subsequently, the normalized principal component is removed from the normalized time series.

After preprocessing to remove the interference of illumination on the pupils, we aim to extract emotional information from the pupil diameter. The temporal dimension of the input samples is downsampled to 60, and the EYE modality with a sample shape of 240 (4s × 60) ×4 (pupil size, and the X and Y coordinates of the left and right eye gaze points) is fed into the eye movement feature extractor (CNN) to extract features. In our eye movement feature extraction model, we construct four convolutional blocks. Each convolutional block consists of a normalization layer and an adaptive pooling layer (performing only max pooling along the temporal dimension). Additionally, each block contains a convolutional layer using ReLU as the activation function and a 1 × 5 convolutional kernel to extract emotional features from eye movements.

### 2.3. Multi-scale feature fusion module

**Figure 5:**
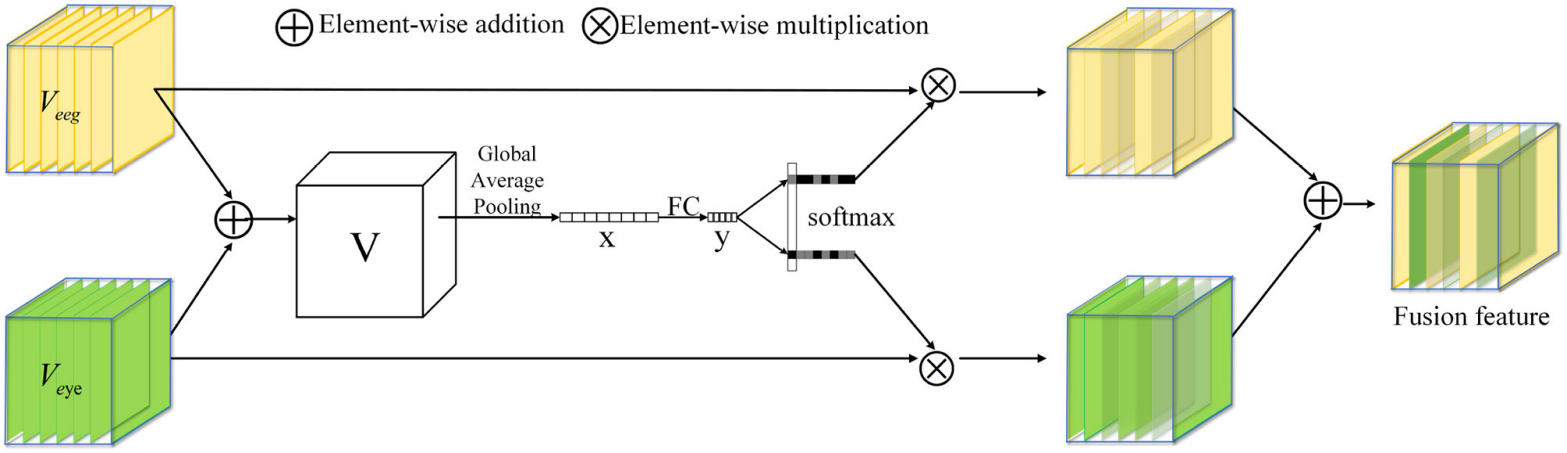
Multi-scale feature fusion (MS Fusion).

In this section, the proposed multi-scale feature fusion method is introduced. Existing feature fusion methods primarily focus on feature selection, but they often overlook the dissimilarity among features, which may limit the model's ability to fully utilize the strengths and weaknesses of different features. To effectively leverage the advantages of different modal features, a multi-scale feature fusion method is designed, as depicted in Figure 5.

In order to discover traits that are more correlated with emotions, efforts are directed toward finding specific traits that show a stronger correlation with emotional states. Through in-depth feature analysis and selection, we find out the features closely related to various emotional expressions. Firstly, the results of the two branches are fused by element-wise summation:


(2) $$\begin{array}{l} V=V_{\text{eeg}}+V_{\text{eye}} \end{array}$$

where *V*_eeg_ and *V*_eye_ represent the characteristics of EEG and Eye mapping, $V_{eeg}\in R^{H\times W\times C}$ and $V_{eye}\in R^{H\times W\times C}$. We then embed global information by simply using global averaging pooling to generate *x* ∈ *R*^*C*^. The purpose of doing this is to extract the global features after fusing the two modalities. Specifically, the *c*-th element of *x* is computed by reducing *V* along the spatial dimensions *H* × *W*:


(3) $$\begin{array}{l} x_{d}=\mathrm{Gap}\left(V_{d}\right)=\frac{1}{H\times W}\overset{H}{\underset{i=1}{\sum }}\overset{W}{\underset{j=1}{\sum }}V_{d}\left(i,j\right) \end{array}$$

Furthermore, we introduce a compact feature *y* ∈ *R*^*d*×1^ to guide accurate and adaptive feature selection. To improve efficiency and reduce dimensionality, we employ a simple fully connected (FC) layer:


(4) y=FC(x)=Relu(fBN(Wx))

where *Relu* function is activation function, *f*_*BN*_ is batch normalization and *W* ∈ *R*^*d*×*C*^. To investigate the impact of *d* on model efficiency, we control its value using a reduction ratio *r*.


(5) $$\begin{array}{l} d=\max\left(C/r,L\right) \end{array}$$

where *L* represents the minimum value of *d* (*L* = 32 is a typical setting in our experiments).

Under the guidance of compact feature *y*, cross-channel soft attention is used to select information of different spatial scales adaptively. The purpose of this is to select the modal features most relevant to emotion, and then we get two weight vectors. Specifically, apply softmax operator on channel numbers:


(6) $$\begin{array}{l} a_{c}=\frac{e^{A_{c}y}}{e^{A_{c}y}+e^{B_{c}y}},b_{c}=\frac{e^{B_{c}y}}{e^{A_{c}y}+e^{B_{c}y}} \end{array}$$

where *A, B* ∈ *R*^*C*×*d*^ and *a*,*b* represent the soft attention vectors for *V*_eeg_ and *V*_eye_. The magnitude of variable *a*,*b* depends on the correlation between the corresponding modality and emotion, with higher correlation leading to larger values. The attention $A_{c}\in R^{1\times d}$ corresponds to the *c*-th row of *A*, *a*_*c*_ refers to the *c*-th element of a, and the same applies to *B*_*c*_ and *b*_*c*_. The final fusion feature is obtained by weighting the attention on each kernel:


(7) $$\begin{array}{l} F_{c}=a_{c}\cdot V_{\text{eeg}}+b_{c}\cdot V_{\text{eye}},a_{c}+b_{c}=1 \end{array}$$

where $F=\left[F_{1},F_{2},\cdots \;,F_{C}\right],F_{c}\in R^{H\times W}$.

After obtaining the fused features, we input them into a fully connected layer and apply the softmax activation function to obtain the classification results. The entire network is trained by minimizing the cross-entropy loss, as shown below:


(8) $$\begin{array}{l} L=-\frac{1}{N}\underset{i}{\sum }\overset{M}{\underset{c=1}{\sum }}y_{ic}\log\left(p_{ic}\right) \end{array}$$

In the equation, *M* represents the total number of categories. The variable *y*_*ic*_ serves as an indicator with binary values (0 or 1), indicating whether the observation sample *i* belongs to category c. Similarly, *p*_*ic*_ represents the predicted probability of the *i*-th observation sample belonging to category c.

## 3. Experiments and discussion

To demonstrate the effectiveness of our MFFNN, we conducted experiments on the SEED-IV dataset and compared it with both single modal and multimodal methods.

### 3.1. Dataset

The SEED-IV dataset, which is a widely used multimodal emotion dataset, was released by Shanghai Jiao Tong University (Zheng et al., 2018). The detailed information of this dataset is presented in Table 1.

**Table 1:** Detailed information about the SEED-IV dataset.

**Attributes**	**Description**
Subject	15 (7 males and 8 females)
Stimulant	72 movie clips
Trials of subjects	72 times (24 × 3)
Length of each trial	125 s (5 s hint of start, 120 s film clip)
Number of electrodes	62 electrodes
Emotional labels	Happy, sad, fearful, neutral
EEG data	Array shape 24 × 62 × data
	(24 experiments, 62 channels)

**Figure 6:**
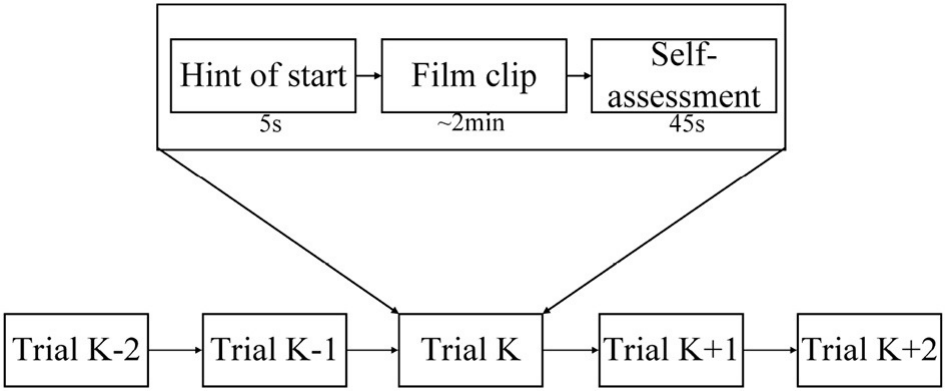
The detailed arrangement of the experiment.

**Figure 7:**
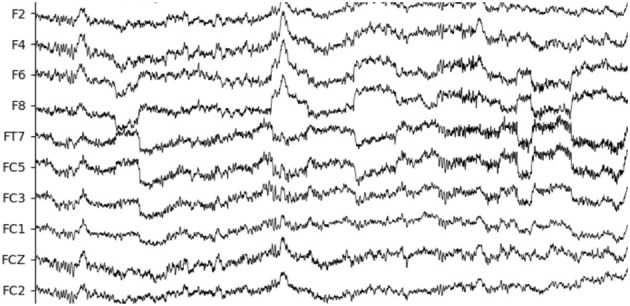
A fragment of EEG signals from selected channels.

**Figure 8:**
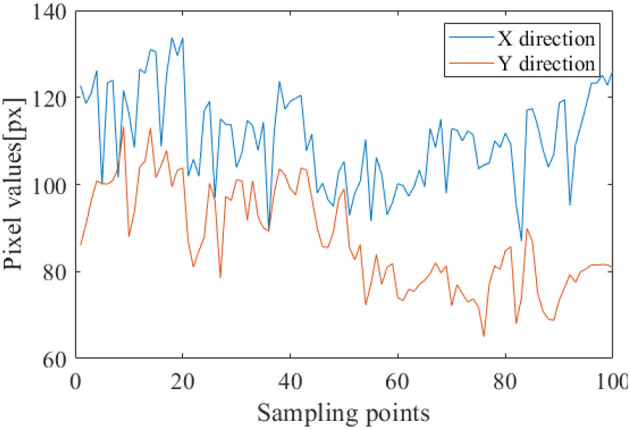
Average pupil size variation curve.

In the SEED-IV dataset induction experiment, 44 participants (22 males, all college students) were recruited to self-evaluate their emotions. A library of 168 movie clips representing four emotions (happiness, sadness, fear, and neutrality) was selected. Through preliminary research, 72 movie clips were carefully chosen, and the experimental procedure was similar to that of SEED. Fifteen participants underwent three sessions of experiments on different days, with each participant watching 6 movie clips per session, resulting in a total of 24 experiments. During the experiments, their EEG signals and eye movement data were simultaneously collected using a 62-channel ESI neuroimaging system and SMI eye movement glasses. The specific experimental procedure is illustrated in Figure 6. Participants watched one of the movie clips, with a 5-second prompt before each segment, and each movie clip lasted approximately two minutes, followed by a 45-second feedback period. Figure 7 displays the raw EEG curves of a participant with certain channels downsampled to 200 Hz during a single experiment. Figure 8 illustrates the variation curves of the average pupil size [px] X and average pupil size [px] Y during the same experiment (blue represents X, orange represents Y).

### 3.2. MFFNN realization

In this study, the proposed method is evaluated on the SEED-IV dataset, which consists of data from 15 participants, each of whom underwent 3 sessions and experienced four emotion types (happiness, sadness, fear, and neutrality). Each participant's session includes 24 experiments, resulting in a total of 1,080 samples. We split the dataset into 80% original training data and 20% test data. To ensure that each fold includes all four different emotion categories, we adopt 5-fold cross-validation in our experiments. The data set partitioning method is consistent with prior studies (Lu et al., 2015; Liu et al., 2019).

**Figure 9:**
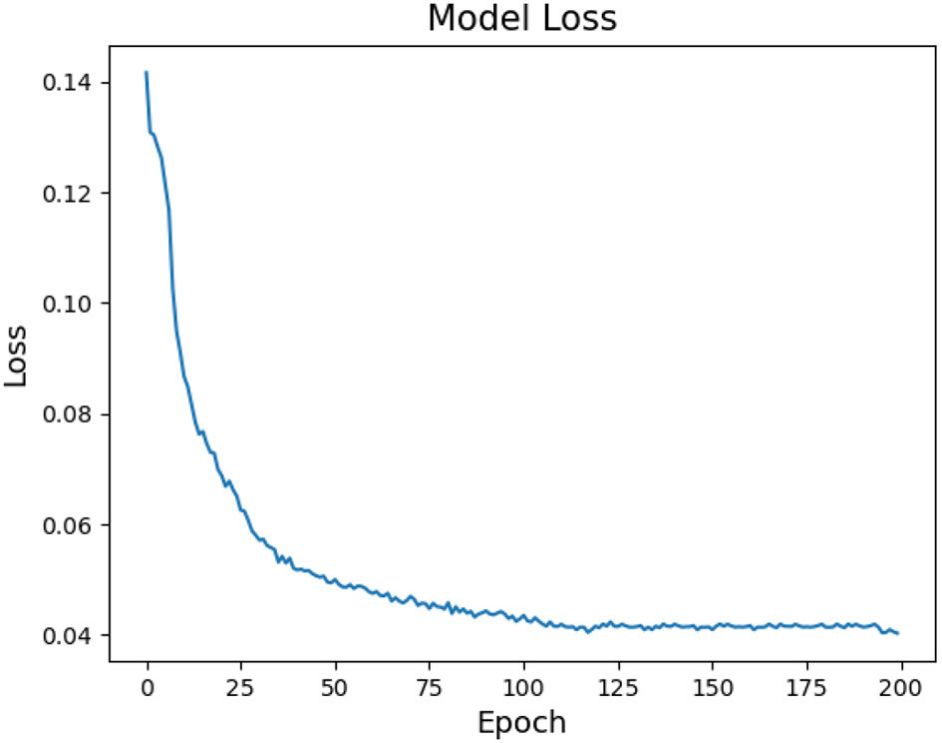
MFFNN training loss changes with epoch.

**Figure 10:**
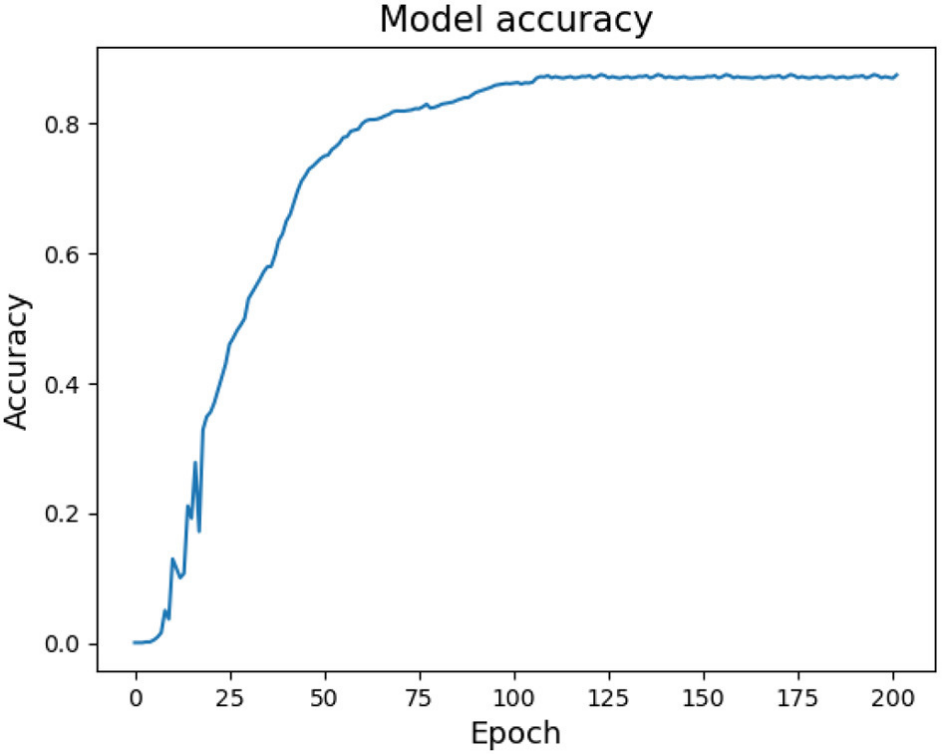
MFFNN training accuracy changes with epoch.

Moreover, this section provides an explanation of the network parameters used in MFFNN, accompanied by an analysis of the impact of specific parameters on the experimental results. The rationale behind the selection of these parameters is discussed. Figures 9, 10 represent the changes in loss and accuracy with epochs during the model's training process. As shown in the figures, when the epoch value reaches around 100 (with minimal deviation), the loss and accuracy gradually converge and essentially stabilize. Thus, in order to achieve better recognition performance, we set the epoch to 100 in the experiments. Table 2 provides the main hyperparameters of the pre-trained MFFNN model, along with their current values or types, and other relevant information.

**Table 2:** The primary hyperparameters for training the MFFNN model.

**Hyperparameters**	**Value**
Emotion categories	4
Downsampling frequency	60
Window size	4s
Learning rate	0.001
Epoch	100
Batch size	256
Dropout	0.2
Hidden layer	256

### 3.3. Experimental results

#### 3.3.1. Comparison with multimodal methods

To demonstrate the superiority of the approach, comparisons are conducted between the proposed MFFNN and other methods reported in the literature. The objective is to substantiate the effectiveness of the proposed model. For the evaluation metrics, we employed accuracy, standard deviation, precision, and F1-score. The calculation formulas are as follows:


(9) $$\begin{array}{c} \text{Accuracy}=\frac{TP+TN}{TP+FN+TN+FP} \end{array}$$

(10) $$\begin{array}{c} \text{precision}=\frac{TP}{TP+FP} \end{array}$$

(11) $$\begin{array}{c} \text{F}1-\text{Score}=\frac{2\cdot precision\cdot recall}{precision+recall} \end{array}$$

where the true positive (TP) and false negative (FN) respectively indicate that the target sample is correctly or incorrectly classified, and the true negative (TN) and false positive (FP) respectively indicate that the non-target sample is correctly or incorrectly classified.

Table 3 presents the comparison results of accuracy, standard deviation, precision, and F1-score between our proposed model and eight other multimodal methods, using EEG and Eye modalities as inputs. Among them, Lu et al. (2015) proposed three methods, namely DLF-SUM, DLF-MAX, and FLF, which combine eye movements and EEG signals to enhance emotion recognition. Zheng et al. (2018) utilized EmotionMeter to integrate EEG and eye movements and employed a bidirectional autoencoder (BDAE) for shared representation extraction to improve recognition performance. Qiu et al. (2018) used DCCA to jointly learn the parameters of nonlinear transformations for two modalities, maximizing their correlation and demonstrating its efficacy in enhancing emotion classification accuracy. Lan et al. (2020) proposed DGCCA-AM, a method that aims to extract emotion-relevant information from multiple modalities while discarding noise. It achieves this by adjusting the weight matrix to maximize the correlation among different modalities, which can be extended to arbitrary modalities. Zhao et al. (2021) improved DCAN by introducing a novel collaborative attention layer that enhances the weights of key feature channels and establishes correlations between different modalities. Zhou et al. (2022) proposed the SOFNN model, treating eye movements as subjective signals and EEG signals as objective signals, effectively learning spatiotemporal information from EEG signals and dynamically integrating EEG and eye movement signals. From Table 3, it can be observed that compared to previous multimodal (EEG and eye movement) methods, although there is not a substantial improvement in accuracy, significant advancements are achieved in precision and F1-score. This directly reflects the superior performance of the proposed method.

**Table 3:** Comparison with multimodal methods.

**Methods**	**ACC (%)**	**STD (%)**	**P (%)**	**F1-Score (%)**
DLF-SUM (Lu et al., 2015)	82.99	9.70	72.51	74.23
FLF (Lu et al., 2015)	83.70	6.92	69.02	70.24
DLF-MAX (Lu et al., 2015)	81.71	6.43	86.84	73.32
EmotionMeter (Zheng et al., 2018)	75.88	16.44	80.24	69.22
DCCA (Qiu et al., 2018)	81.74	9.23	88.97	77,21
DGCCA-AM (Lan et al., 2020)	82.11	2.76	84.15	76.62
mDCAN (Zhao et al., 2021)	85.04	6.62	89.37	81.56
SOFNN (Zhou et al., 2022)	86.27	10.16	90.72	80.83
MFFNN (Our model)	87.32	6.41	94.32	85.04

**Figure 11:**
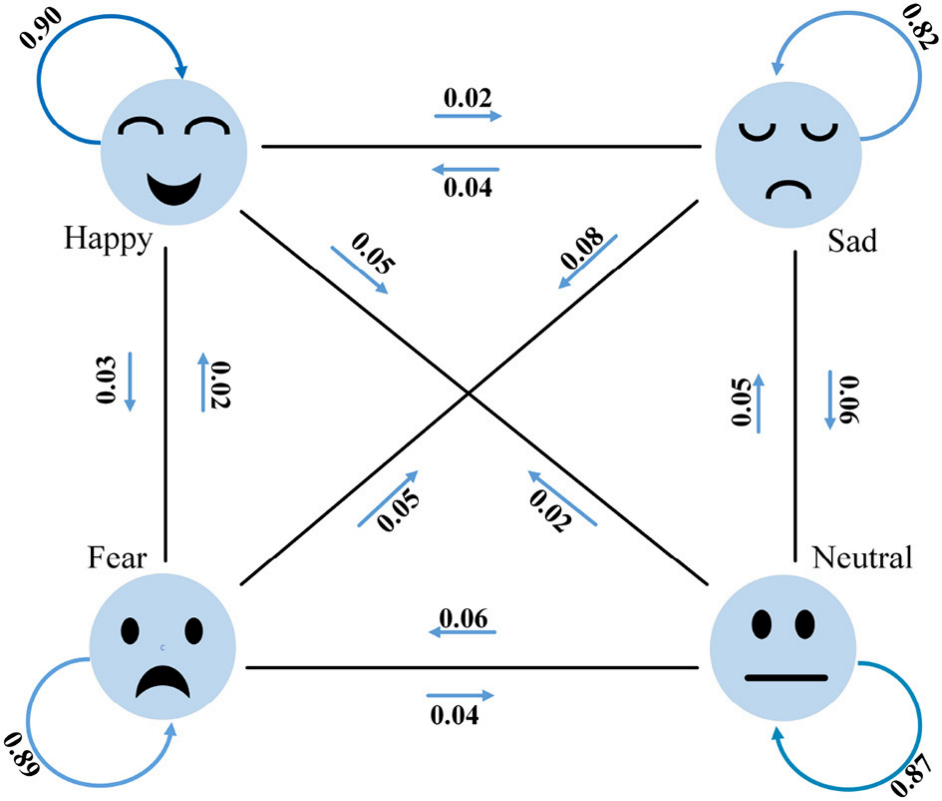
Confusion graph of MFFNN.

To facilitate a better understanding of the emotion classification performance of MFFNN, we generate a confusion matrix for MFFNN, as shown in Figure 11. The numbers in the figure represent the accuracy rates for each class. From the figure, it can be observed that the MFFNN model performs well in classifying happy and fearful emotions (with accuracy rates of 90 and 89% respectively), while its recognition performance for sad emotions is relatively poorer (only 82%). Furthermore, it is evident from the figure that similar emotions are more prone to confusion. For instance, 8% of the sad emotions are misclassified as fearful, and 6% of the sad emotions are misclassified as neutral. Additionally, neutral emotions are the most easily confused. These findings align with our experimental expectations and validate the rationale behind our experimental design.

#### 3.3.2. Comparison with EEG-based methods

In order to demonstrate the effectiveness of the proposed MFFNN compared to existing EEG-based emotion recognition methods, further comparisons were made with single-modal EEG approaches. The comparative results are presented in Table 4. It is evident that traditional SVM (Wang et al., 2011) methods are outperformed by deep learning approaches. Among the deep learning methods (Jia et al., 2020; Wang et al., 2021; Zhang et al., 2023), the MFFNN model exhibits comparable performance. With higher accuracy (87.32%), precision (94.32%), and F1-score (85.04%) on the task compared to other methods, the MFFNN model demonstrates superior performance. This finding suggests that the MFFNN model is capable of leveraging complementary features between the two modalities and utilizing their complementarity for effective emotion classification. It also validates the advantages of the proposed approach in comparison to single-modal (EEG) emotion recognition.

**Table 4:** Comparison with EEG modal methods.

**Methods**	**Modality**	**ACC (%)**	**STD (%)**	**P (%)**	**F1-Score (%)**
SVM (Wang et al., 2011)	EEG	56.61	20.05	76.94	52.54
DBN (Zheng and Lu, 2015)	EEG	66.77	7.38	78.48	60.24
CRNN-DF (Zhang et al., 2023)	EEG	72.74	10.44	80.18	64.36
CNN-R (Manor and Geva, 2015)	EEG	73.99	3.94	85.71	71.27
BiHDM (Li et al., 2018)	EEG	74.35	74.35	84.94	69.70
Shallow ConvNet (Schirrmeister et al., 2017)	EEG	76.74	4.19	87.22	70.61
RGNN (Zhong et al., 2020)	EEG	79.37	10.54	89.73	71.97
EEGNet (Lawhern et al., 2018)	EEG	79.12	2.05	91.20	73.32
ERP-CapsNet (Ma et al., 2021)	EEG	80.49	3.34	90.67	73.49
MS-CNN (Wang et al., 2021)	EEG	83.00	2.32	93.74	75.21
Transformer (Liu et al., 2021)	EEG	83.27	8.37	92.31	78.64
SST-EmotionNet (Jia et al., 2020)	EEG	84.92	6.66	94.28	80.83
MFFNN	EEG, Eye	87.32	6.41	94.32	85.04

### 3.4. Model analysis of MFFNN

**Figure 12:**
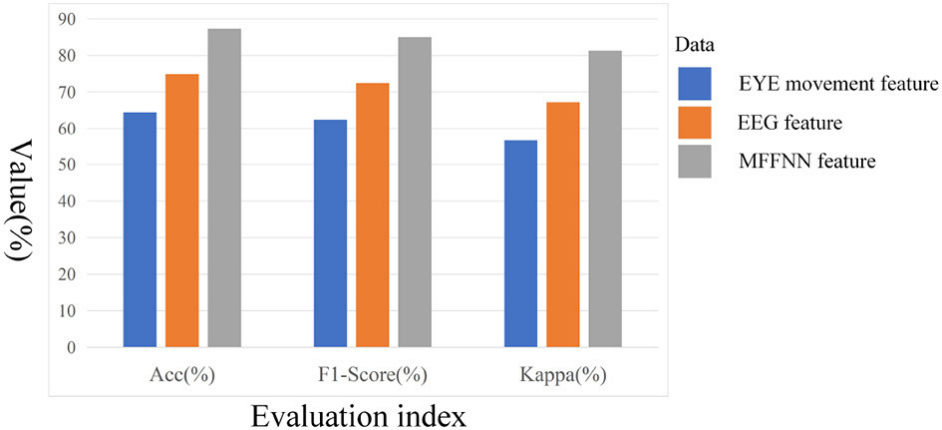
The effect of different characteristics on emotional classification.

To validate the effectiveness of the model in feature extraction and multimodal feature fusion, experiments were designed to analyze the roles and effects of different modules. Firstly, the effectiveness of the dual-branch feature extraction module was verified. Specifically, EEG signal features were extracted as described in this paper, and a softmax classifier was used for training this model using the EEG signal data from the SEED-IV dataset. Additionally, separate training was conducted for the eye movement signal features, also using softmax as the classifier. The experimental results are presented in Table 5 and Figure 12. F1-score was employed to evaluate whether the model had any emotion omissions in the case of a limited number of samples for the SEED-IV dataset. Furthermore, the Kappa statistic was used to ensure that the model did not exhibit bias when applied to different emotion datasets with significant variations in sample size.

**Table 5:** Comparative analysis of models.

**Methods**	**ACC (%)**	**STD (%)**	**F1-Score (%)**	**Kappa (%)**
Eye	64.42	12.62	62.39	56.61
EEG	74.88	10.06	72.47	67.22
MFFNN	87.32	6.41	85.04	81.31

From Table 5 and Figure 12, it can be observed that the accuracy of both models mentioned above is not very high, with the accuracy of the EEG signal being higher than that of the eye movement signal. This indicates that subjective behavioral signals are influenced by various factors, while objective physiological signals are less susceptible to deception. Additionally, it suggests that a single modal signal alone is insufficient to accurately describe emotions, which further confirms our previous statement. Compared to traditional recognition methods, the dual-branch feature extraction module, which is used solely for extracting emotional information from the two modalities, has demonstrated good performance. This, to some extent, also confirms the feasibility of this module.

After validating the dual-branch feature extraction module, the study further examines the multi-scale feature fusion module. By comparing the aforementioned two models with MFFNN, the role of the multi-scale feature fusion module in the network is explored. As shown in Table 5 and Figure 12, compared to the sole use of EEG and eye movement signals, the MFFNN model achieves better results (87.32% accuracy). The F1-score and kappa also show significant improvements, indicating that the MFFNN model can perform well even with varying sample sizes, demonstrating better model adaptability.

**Figure 13:**
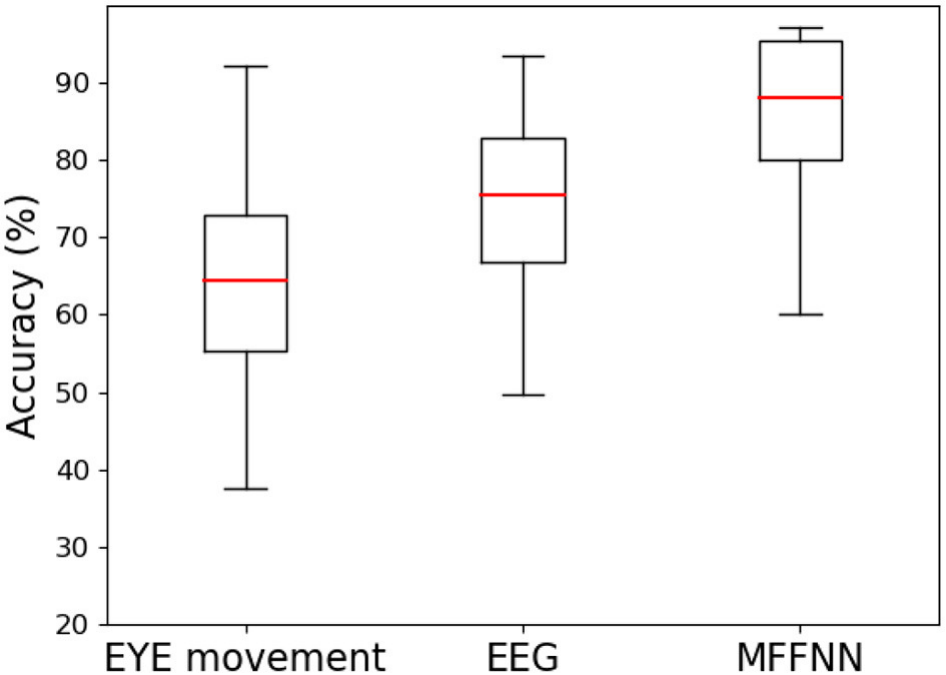
Precision box diagrams with different features.

Figure 13 measures the accuracy distribution and fluctuation obtained from different features. From the figure, it can be observed that the accuracy solely based on eye movement signals is the lowest and exhibits significant fluctuations, which is closely related to individual differences. Compared to the two single modal approaches, the MFFNN method achieves the best performance and enhances the robustness of the model.

**Figure 14:**
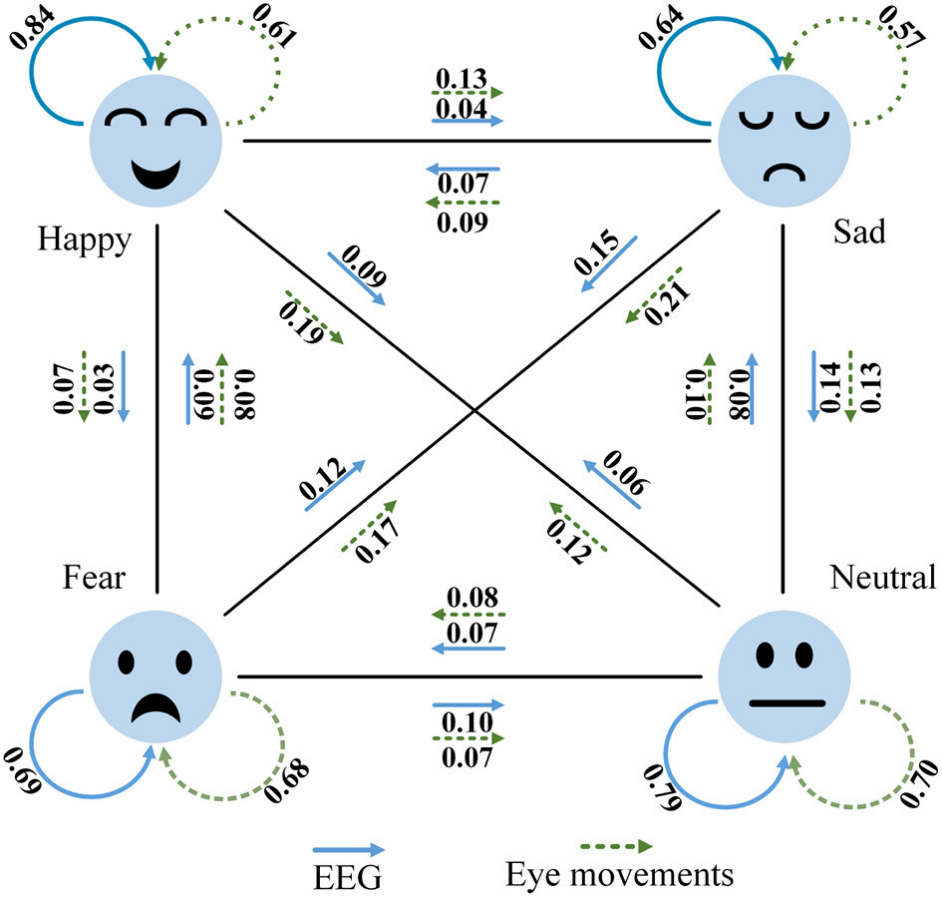
EEG and eye movement confusion (Solid blue arrows are EEG signals and dotted green eye movement signals).

Figure 14 depicts the classification performance of EEG and eye movement signals for different emotions. From the graph, it can be observed that EEG signals exhibit better recognition performance for the emotion of happiness (84%), while eye movement signals perform better in recognizing neutral emotions (70%). Additionally, we noticed significant variations in the recognition performance of each emotion by individual signals. For instance, EEG signals achieve a recognition rate of 84% for happiness but only 64% for sadness. Eye movement signals achieve a recognition rate of 70% for neutrality but only 57% for sadness. We also found that EEG signals have a 14% probability of misclassifying sadness as neutrality. On the other hand, eye movement signals show higher accuracy in recognizing neutrality and sadness but are more prone to confusion between sadness and fear. EEG signals perform relatively better in this aspect compared to eye movement signals, which validates the complementary nature of the two modalities. Comparing Figure 14 with Figure 11, it is evident that the MFFNN framework, which integrates EEG and eye movement signals, significantly improves the recognition performance for different emotions and reduces the probability of confusion. This comparison confirms that the proposed MFFNN framework effectively exploits the emotional features of both EEG and eye movement modalities, utilizing their complementarity to enhance the accuracy of emotion recognition.

**Figure 15:**
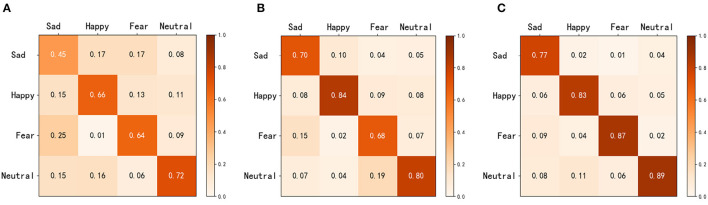
Confusion matrices with different characteristics [**(A)** represents eye movement feature, **(B)** represents EEG feature, and **(C)** represents MFFNN feature].

We also present the confusion matrices for the three aforementioned methods, as shown in Figure 15. In Figures 15A–C represent eye movement features, EEG features, and MFFNN, respectively. The horizontal axis represents the actual labels of the stimuli, while the vertical axis represents the emotion labels obtained after classification by the network. From the figure, it is evident that both eye movement features and EEG features result in confusion between different emotions. In contrast, our MFFNN exhibits superior performance in emotion recognition compared to the single modal methods. The MFFNN is capable of assigning different weights to the two modal features based on their correlation with emotions in various emotion allocation tasks, thereby fully exploiting the complementarity of the two modal features and improving the accuracy of multimodal emotion recognition.

## 4. Conclusions

In this study, a multimodal feature fusion framework based on MFFNN is proposed. The dual-branch feature extraction module effectively captures essential emotional information from raw EEG and eye movement signals. The multi-scale feature fusion module analyzes the complementarity of the two modal features at different scales, leading to accurate emotion classification. Additionally, a cross-channel soft attention mechanism is employed to selectively emphasize information from different spatial scales, focusing on the modal features most relevant to emotions. The proposed MFFNN framework is validated on the SEED-IV dataset. Through comparisons with single modal and multimodal methods, the multi-scale feature fusion in our approach extensively exploits the complementary characteristics of the two modalities, resulting in enhanced accuracy of emotion recognition compared to single modal approaches. Furthermore, the experiments in this study only considered the four common emotions. However, in practical applications, a broader range of emotions should be taken into account. In future research, the scope of multimodal fusion can be further expanded by integrating more perceptual modalities and sensor data into the emotion recognition framework. In addition to EEG and eye movement signals, other physiological signals such as heart rate and skin conductance, as well as information from modalities like speech, images, and videos, can also be considered. By synthesizing diverse perceptual information, we can gain a more comprehensive understanding of an individual's physiological and psychological responses in different emotional states, thereby further enhancing the accuracy and reliability of emotion recognition.

## Data availability statement

The original contributions presented in the study are included in the article/supplementary material, further inquiries can be directed to the corresponding author.

## Author contributions

BF: conceptualization, methodology, software, investigation, formal analysis, and writing—original draft. CG: data curation. MF: visualization and investigation. YX: resources and supervision. YL: conceptualization, funding acquisition, resources, supervision, and writing—review and editing. All authors contributed to the article and approved the submitted version.
