# Corrigendum: A novel feature fusion network for multimodal emotion recognition from EEG and eye movement signals

**DOI:** 10.3389/fnins.2023.1287377

**Published:** 2023-09-25

**Authors:** Baole Fu, Chunrui Gu, Ming Fu, Yuxiao Xia, Yinhua Liu

**Affiliations:** ^1^School of Automation, Qingdao University, Qingdao, China; ^2^Institute for Future, Qingdao University, Qingdao, China; ^3^Shandong Key Laboratory of Industrial Control Technology, Qingdao, China

**Keywords:** multimodal emotion recognition, electroencephalogram (EEG), eye movement, feature fusion, multi-scale, Convolutional Neural Networks (CNN)

In the published article, there was an error in the legend for “Figure 1. Multimodal emotion recognition framework. (A) Dual branch feature extraction module. (B) Multi-scale feature fusion module.” as published. There are a few errors in the comments in the diagram, one is the convolutional block word error, all ConvBlovk is changed to ConvBlock. The following four EEGConvBlovk should be replaced with EYEConvBlock. The correct legend appears below.

**Figure 1:**
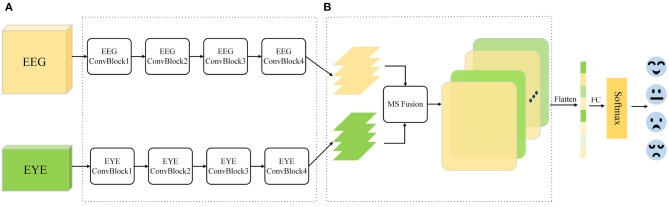
Multimodal emotion recognition framework. **(A)** Dual branch feature extraction module. **(B)** Multi-scale feature fusion module.

The authors apologize for this error and state that this does not change the scientific conclusions of the article in any way. The original article has been updated.

